# Supplementary material for: Quadrupolar ordering and exotic magnetocaloric effect in RB4 (R = Dy, Ho)
Source: Sci Rep. 2020 Jan 21;10:803. doi: 10.1038/s41598-020-57621-7 (PMC6972750; doi:10.1038/s41598-020-57621-7)
Supplement: Supplementary file 1 — Supplementary information. [file 41598_2020_57621_MOESM1_ESM.docx]

Quadrupolar ordering and exotic magnetocaloric effect in *R*B_4_ (*R* = Dy, Ho)

M.S. Song^1^, K. K. Cho^1^, B.Y. Kang^1^, S. B. Lee^2^, B. K. Cho^1*^*^1^School of Materials Science and Engineering, Gwangju Institute of Science and Technology (GIST), Gwangju 61005, Korea*

*^2^Department of Physics, Korea Advanced Institute of Science and Technology (KAIST), Daejeon, 34141, Korea*

^*^E-mail: chobk@gist.ac.kr


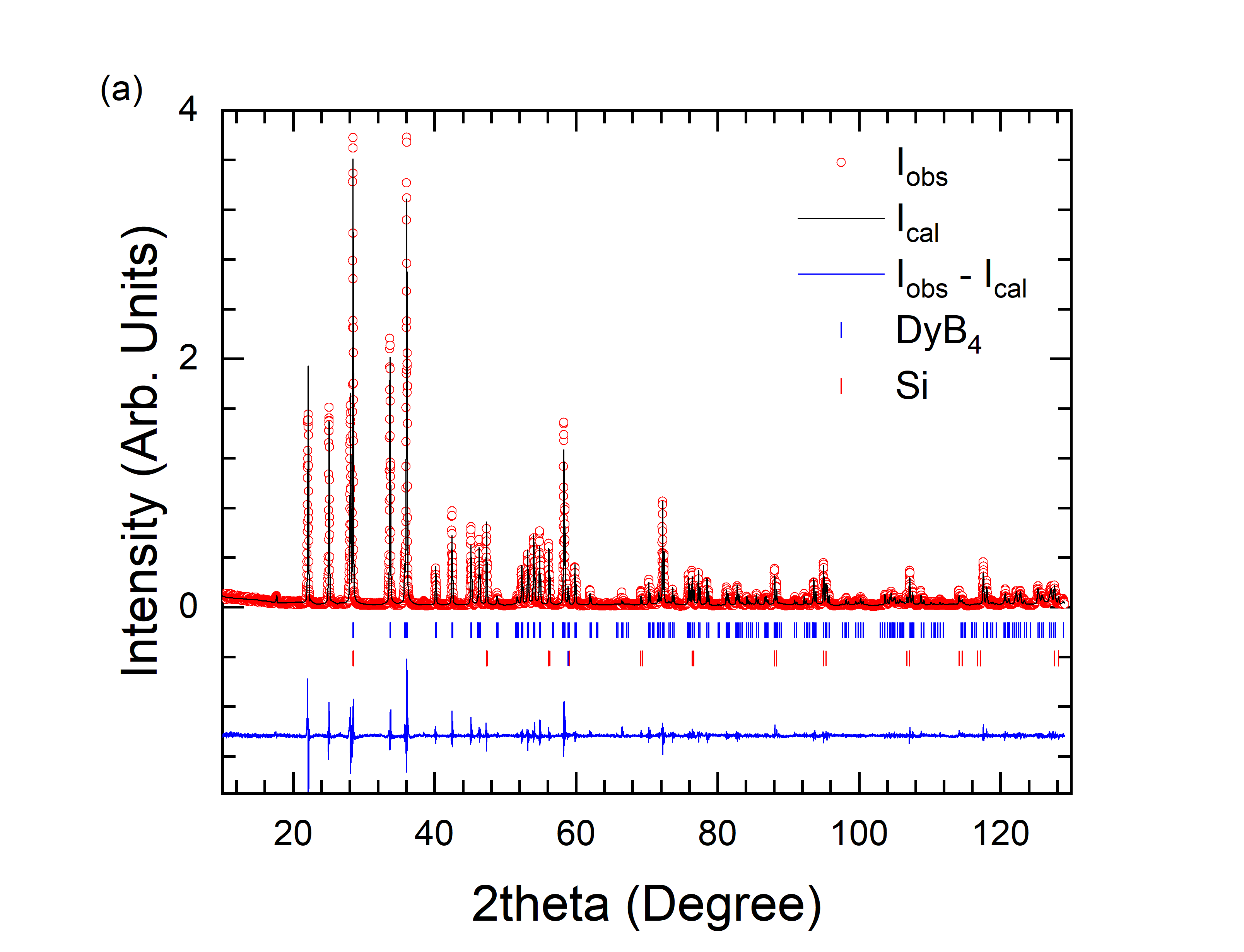

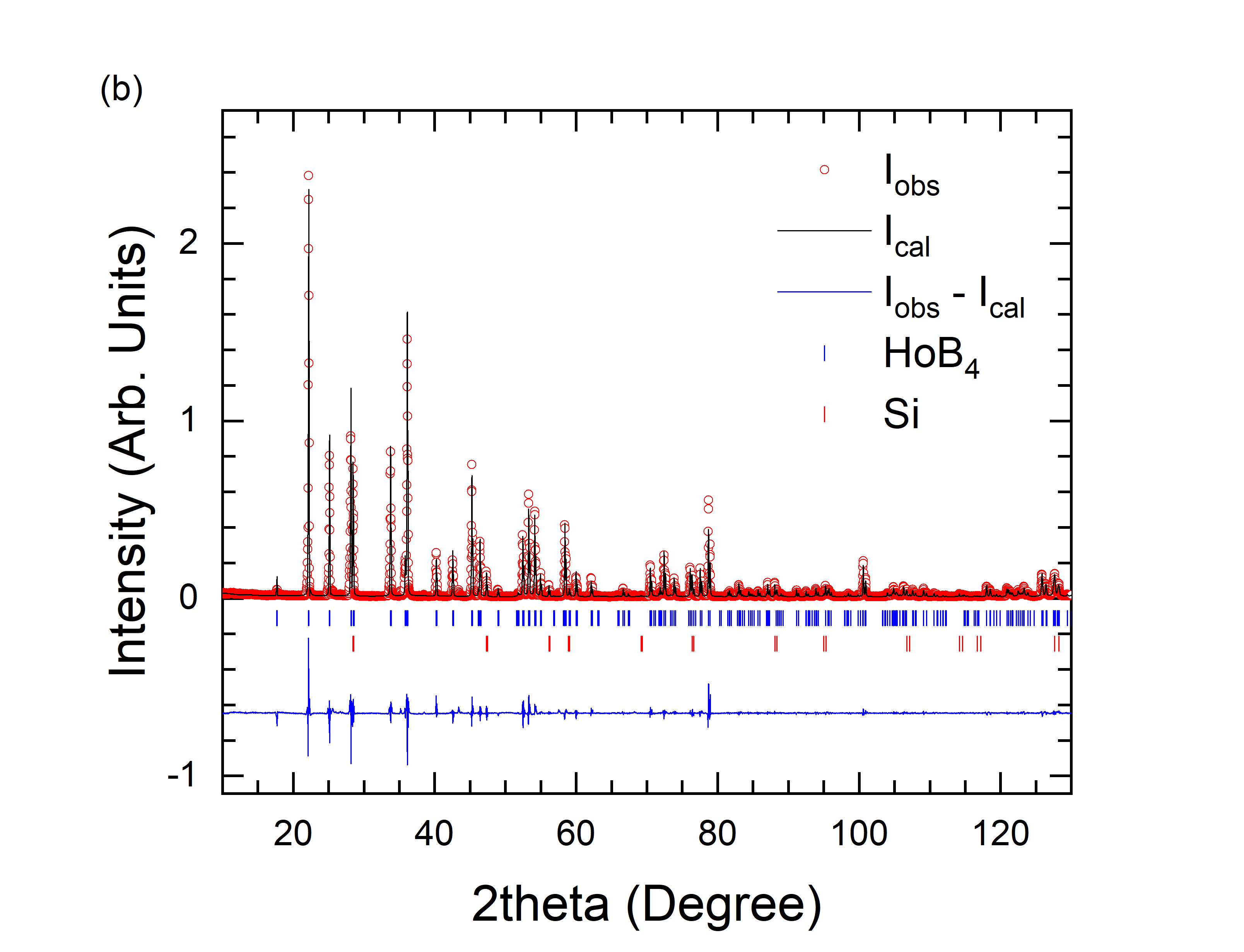


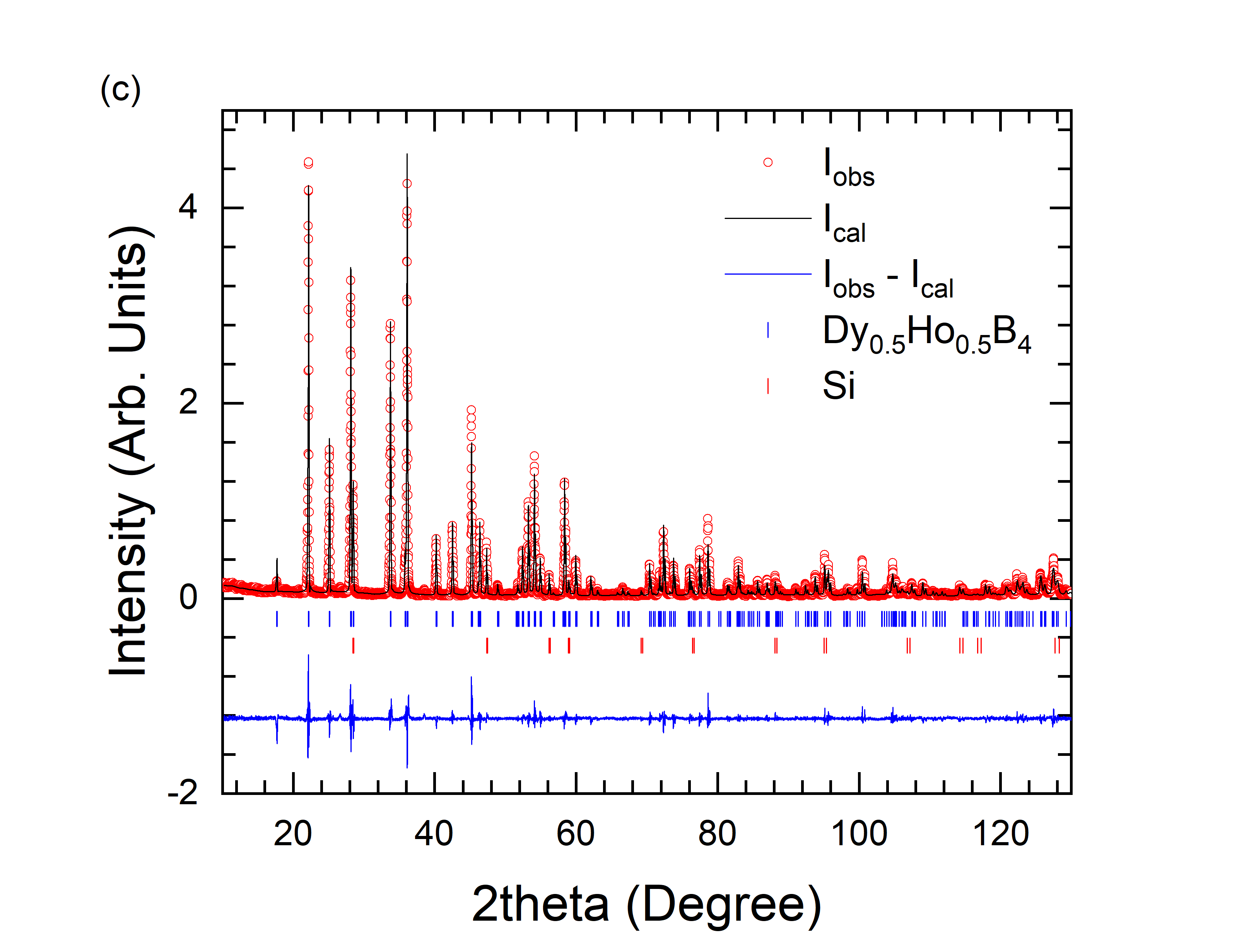


Supplementary Figure 1: Powder X-ray diffraction patterns of DyB_4_, HoB_4_ and Dy_0.5_H_o0.5_B_4_. The black solid line is the theoretical diffraction pattern, which is obtained using FULLPROF. The blue and red vertical bars are the Bragg peak positions of the samples and of Si, respectively. The lowest curves represent the difference between the experimental and calculated intensities.

|  | a (Å) | b (Å) | c (Å) | *T*_N2_ (K) | *T*_N1_ (K) |
| --- | --- | --- | --- | --- | --- |
| DyB_4_ | 7.099(5) | 7.099(5) | 4.015(9) | 13 | 20.1 |
| Dy_0.5_Ho_0.5_B_4_ | 7.088(4) | 7.088(4) | 4.008(7) | 9.5 | 13.8 |
| HoB_4_ | 7.083(8) | 7.083(8) | 4.005(1) | 5.5 | 7.5 |

Supplementary Table 1: Refined lattice parameters of Ho_1-_*_x_*Dy*_x_*B_4_ (*x* = 0.0, 0.1, and 1.0).

Supplementary Note 1.

The powder X-ray diffraction patterns are collected for the pulverized single crystals. The patterns show a single phase without any noticeable impurities. The crystal structure is consistent with the tetragonal symmetry of the ThB4-type structure with the space group *P4/mbm* (#127) at room temperature. The lattice constants vary, and the volume decreases with an increasing Ho concentration in Ho_1-_*_x_*Dy*_x_*B_4_. The lattice parameters and volume follow Vegard’s law. The antiferromagnetic transition temperature is also observed to systematically decrease as the Ho concentration increases, which indicates a uniform distribution of both Ho and Dy in Ho_1-_*_x_*Dy*_x_*B_4_.


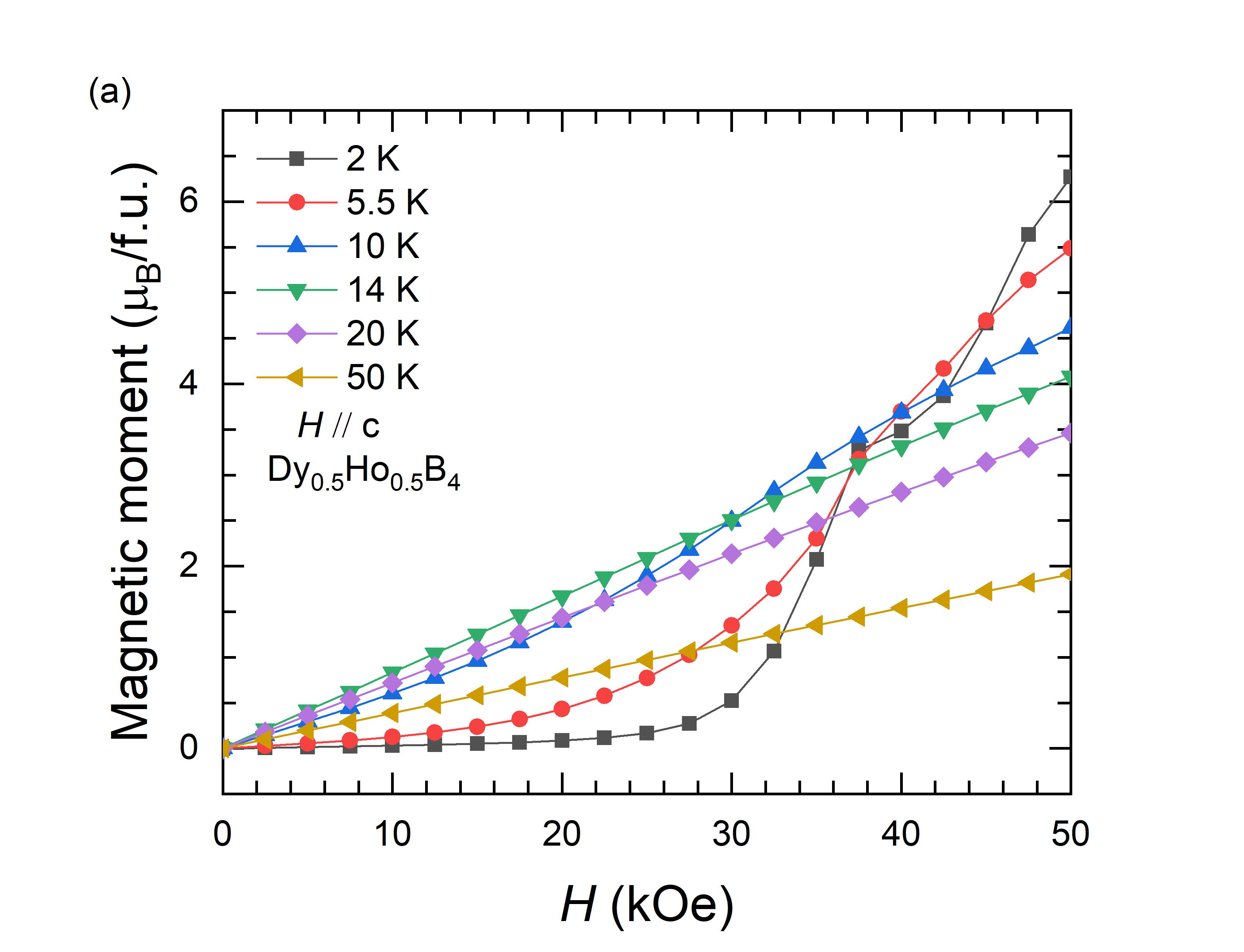

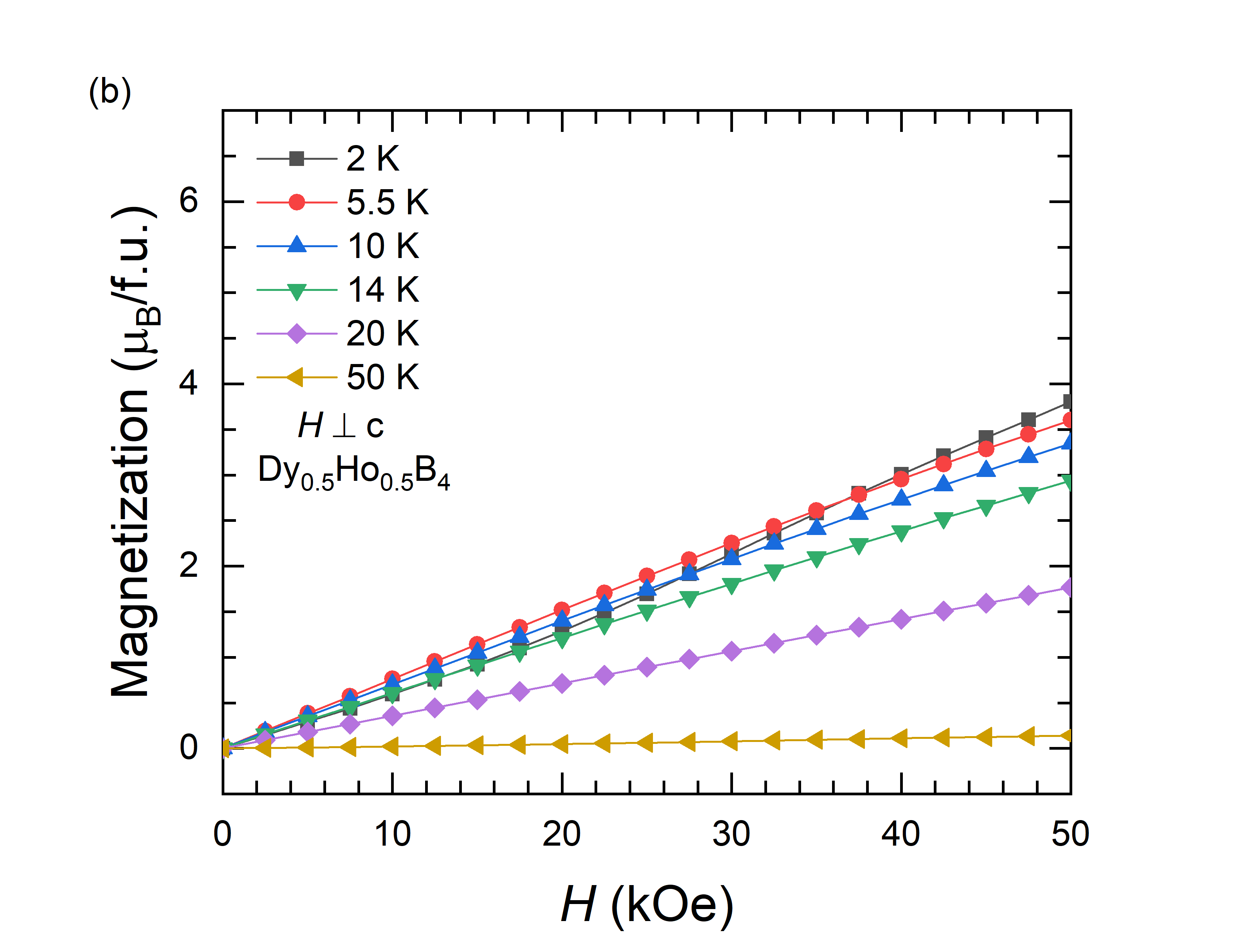


Supplementary Figure 2: Magnetic field dependence of the isothermal magnetization at different temperatures in a range of 2 K ≤ *T* ≤ 50 K along the *c*-axis (a) and *ab* plane (b) for Dy_0.5_Ho_0.5_B_4_.


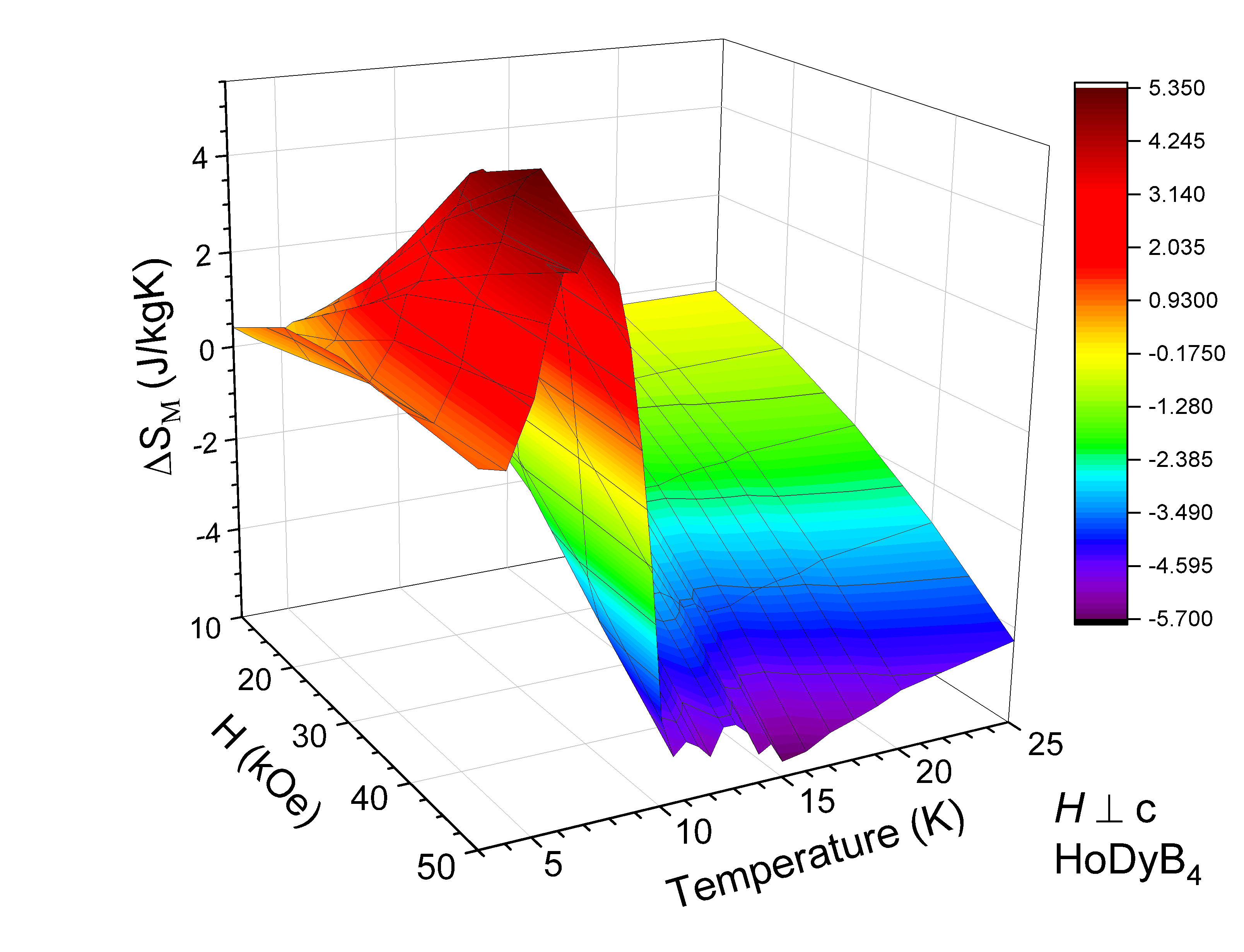


Supplementary Figure 3: Temperature-dependence of the magnetic entropy change of Dy_0.5_Ho_0.5_B_4_ during different magnetic fields of 10, 20, 30, 40 and 50 kOe along the *ab* plane.

Supplementary Reference
[1] B.Y. Kang, S. S. Lee, M. S. Song, K. K. Cho, S. H. Han, B. K. Cho, Curr. Appl. Phys. 16, 1001 (2016)

asfdlkjasldf

Supp

sdf

sdsdfsdafasdfasdfsd
